# Supplementary material for: Hot spot prediction in protein-protein interactions by an ensemble system
Source: BMC Syst Biol. 2018 Dec 31;12(Suppl 9):132. doi: 10.1186/s12918-018-0665-8 (PMC6311905; doi:10.1186/s12918-018-0665-8)
Supplement: Supplementary file 2 — All relevant MATLAB code. A simple Matlab implement of our predictor. (ZIP 19,742 kb) [file 12918_2018_665_MOESM2_ESM.zip › Additional file 2/Read ASA/ASA Property/SKEMPI/2WPTA.txt]

 NetSurfP - Protein Surface Accessibility and Secondary Structure Predictions

  

|  |  |  |
| --- | --- | --- |
|  |  | NetSurfP - Protein Surface Accessibility and Secondary Structure Predictions  Technical University of Denmark |

  


---

```
# For publication of results, please cite:
# A generic method for assignment of reliability scores applied to solvent accessibility predictions.
# Bent Petersen, Thomas Nordahl Petersen, Pernille Andersen, Morten Nielsen and Claus Lundegaard
# BMC Structural Biology 2009, 9:51 doi:10.1186/1472-6807-9-51
#
# Column 1: Class assignment - B for buried or E for Exposed - Threshold: 25% exposure, but not based on RSA
# Column 2: Amino acid
# Column 3: Sequence name
# Column 4: Amino acid number
# Column 5: Relative Surface Accessibility - RSA
# Column 6: Absolute Surface Accessibility
# Column 7: Z-fit score for RSA prediction
# Column 8: Probability for Alpha-Helix
# Column 9: Probability for Beta-strand
# Column 10: Probability for Coil
E K  2WPTA                  1    0.840 172.850   1.121   0.003   0.003   0.994
E H  2WPTA                  2    0.583 106.120  -0.429   0.115   0.016   0.868
E S  2WPTA                  3    0.533  62.468   1.108   0.181   0.016   0.803
B I  2WPTA                  4    0.173  32.005  -1.024   0.430   0.016   0.555
E S  2WPTA                  5    0.453  53.045  -0.377   0.339   0.016   0.645
E D  2WPTA                  6    0.504  72.626  -0.163   0.339   0.016   0.645
B Y  2WPTA                  7    0.210  44.941   0.932   0.339   0.016   0.645
E T  2WPTA                  8    0.330  45.729   0.529   0.181   0.016   0.803
B E  2WPTA                  9    0.250  43.710   0.423   0.858   0.002   0.139
E A  2WPTA                 10    0.609  67.134   0.455   0.923   0.002   0.076
E E  2WPTA                 11    0.307  53.546   1.336   0.970   0.001   0.030
B F  2WPTA                 12    0.030   6.101   0.597   0.988   0.000   0.012
B L  2WPTA                 13    0.170  31.219   1.412   0.988   0.000   0.012
E E  2WPTA                 14    0.481  83.978   1.434   0.988   0.000   0.012
B F  2WPTA                 15    0.070  14.009   0.768   0.988   0.000   0.012
B V  2WPTA                 16    0.026   4.073   0.824   0.988   0.000   0.012
E K  2WPTA                 17    0.371  76.376   1.546   0.988   0.000   0.012
E K  2WPTA                 18    0.402  82.774   1.792   0.970   0.001   0.030
B I  2WPTA                 19    0.029   5.439   0.375   0.970   0.001   0.030
B A  2WPTA                 20    0.116  12.761   0.581   0.938   0.007   0.055
E R  2WPTA                 21    0.601 137.537   1.363   0.694   0.003   0.303
E A  2WPTA                 22    0.372  40.994  -0.244   0.321   0.003   0.675
E E  2WPTA                 23    0.646 112.926  -1.261   0.109   0.005   0.886
E G  2WPTA                 24    0.587  46.197  -1.358   0.016   0.005   0.979
E A  2WPTA                 25    0.445  49.061  -0.215   0.016   0.005   0.979
E T  2WPTA                 26    0.469  65.064  -0.304   0.016   0.005   0.979
E E  2WPTA                 27    0.551  96.190   0.005   0.246   0.004   0.750
E C  2WPTA                 28    0.557  78.161  -0.358   0.502   0.002   0.495
E D  2WPTA                 29    0.385  55.536   0.056   0.694   0.003   0.303
B D  2WPTA                 30    0.215  30.967  -0.242   0.858   0.002   0.139
E N  2WPTA                 31    0.349  51.123  -0.670   0.858   0.002   0.139
E K  2WPTA                 32    0.518 106.511   1.500   0.923   0.002   0.076
B L  2WPTA                 33    0.031   5.694   0.408   0.970   0.001   0.030
B V  2WPTA                 34    0.056   8.669   1.044   0.970   0.001   0.030
E R  2WPTA                 35    0.489 111.981   1.356   0.970   0.001   0.030
B E  2WPTA                 36    0.233  40.670   1.387   0.970   0.001   0.030
B F  2WPTA                 37    0.023   4.656   1.154   0.970   0.001   0.030
E E  2WPTA                 38    0.377  65.775   1.486   0.923   0.002   0.076
E R  2WPTA                 39    0.466 106.714   1.801   0.938   0.007   0.055
B L  2WPTA                 40    0.044   8.020   0.845   0.802   0.014   0.185
B T  2WPTA                 41    0.128  17.781   0.140   0.660   0.049   0.291
E E  2WPTA                 42    0.518  90.530   0.288   0.184   0.043   0.773
B H  2WPTA                 43    0.161  29.250   1.040   0.058   0.017   0.925
B P  2WPTA                 44    0.225  31.871  -0.057   0.018   0.019   0.964
E D  2WPTA                 45    0.610  87.887  -0.476   0.018   0.019   0.964
E G  2WPTA                 46    0.377  29.701  -1.607   0.018   0.019   0.964
B S  2WPTA                 47    0.330  38.617  -1.126   0.018   0.019   0.964
B D  2WPTA                 48    0.216  31.183  -1.081   0.004   0.085   0.910
B L  2WPTA                 49    0.043   7.818   0.553   0.004   0.616   0.381
B I  2WPTA                 50    0.041   7.677   0.278   0.002   0.816   0.182
B Y  2WPTA                 51    0.071  15.237  -0.007   0.002   0.816   0.182
B Y  2WPTA                 52    0.150  31.970   1.507   0.004   0.514   0.481
B P  2WPTA                 53    0.076  10.756   0.240   0.004   0.138   0.858
E R  2WPTA                 54    0.653 149.651   0.727   0.018   0.019   0.964
E D  2WPTA                 55    0.743 107.037   0.170   0.016   0.005   0.979
E D  2WPTA                 56    0.604  86.979  -1.397   0.016   0.005   0.979
E R  2WPTA                 57    0.623 142.736  -1.655   0.018   0.019   0.964
E E  2WPTA                 58    0.624 109.013  -0.821   0.018   0.019   0.964
E D  2WPTA                 59    0.273  39.325  -1.730   0.018   0.019   0.964
E S  2WPTA                 60    0.239  28.069   0.531   0.053   0.005   0.942
B P  2WPTA                 61    0.141  20.022  -1.014   0.502   0.002   0.495
E E  2WPTA                 62    0.447  78.126   0.562   0.694   0.003   0.303
E G  2WPTA                 63    0.237  18.660   0.422   0.858   0.002   0.139
B I  2WPTA                 64    0.030   5.606   0.635   0.938   0.007   0.055
B V  2WPTA                 65    0.063   9.698   0.675   0.975   0.003   0.022
E K  2WPTA                 66    0.492 101.287   1.461   0.975   0.003   0.022
B E  2WPTA                 67    0.151  26.292   1.439   0.970   0.001   0.030
B I  2WPTA                 68    0.030   5.457   1.065   0.970   0.001   0.030
E K  2WPTA                 69    0.357  73.435   1.502   0.970   0.001   0.030
E E  2WPTA                 70    0.521  90.984   2.009   0.970   0.001   0.030
B W  2WPTA                 71    0.072  17.268   0.884   0.970   0.001   0.030
B R  2WPTA                 72    0.221  50.586   1.074   0.970   0.001   0.030
E A  2WPTA                 73    0.632  69.679   0.438   0.923   0.002   0.076
E A  2WPTA                 74    0.623  68.688   0.110   0.694   0.003   0.303
E N  2WPTA                 75    0.512  74.913   0.374   0.321   0.003   0.675
E G  2WPTA                 76    0.620  48.770  -1.733   0.053   0.005   0.942
E K  2WPTA                 77    0.340  69.959   0.829   0.016   0.005   0.979
E S  2WPTA                 78    0.566  66.300  -0.742   0.016   0.005   0.979
E G  2WPTA                 79    0.309  24.350  -1.480   0.018   0.019   0.964
B F  2WPTA                 80    0.173  34.781  -2.075   0.058   0.017   0.925
E K  2WPTA                 81    0.582 119.615   0.689   0.058   0.017   0.925
E Q  2WPTA                 82    0.886 158.275   0.831   0.003   0.003   0.994

Explain the output.  Go back.
```

---
